# Genome and evolution of Tibet orbivirus, TIBOV (genus *Orbivirus*, family *Reoviridae*)

**DOI:** 10.3389/fcimb.2024.1327780

**Published:** 2024-03-05

**Authors:** Tingting Gao, Minghua Li, Hong Liu, Shihong Fu, Huanyu Wang, Guodong Liang

**Affiliations:** ^1^ Shandong Provincial Research Center for Bioinformatic Engineering and Technique, School of Life Sciences and Medicine, Shandong University of Technology, Zibo, China; ^2^ National Key Laboratory of Intelligent Tracking and Forecasting for Infectious Diseases, National Institute for Viral Disease Control and Prevention, Chinese Center for Disease Control and Prevention, Beijing, China

**Keywords:** Tibet orbivirus, *Reoviridae*, *Orbivirus*, viral genome structure, molecular genetics, phylogeny

## Abstract

Tibet orbivirus (TIBOV) was first isolated from *Anopheles maculatus* mosquitoes in Xizang, China, in 2009. In recent years, more TIBOV strains have been isolated in several provinces across China, Japan, East Asia, and Nepal, South Asia. Furthermore, TIBOVs have also been isolated from *Culex* mosquitoes, and several midge species. Additionally, TIBOV neutralizing antibodies have been detected in serum specimens from several mammals, including cattle, sheep, and pigs. All of the evidence suggests that the geographical distribution of TIBOVs has significantly expanded in recent years, with an increased number of vector species involved in its transmission. Moreover, the virus demonstrated infectivity towards a variety of animals. Although TIBOV is considered an emerging orbivirus, detailed reports on its genome and molecular evolution are currently lacking. Thus, this study performed the whole-genome nucleotide sequencing of three TIBOV isolates from mosquitoes and midges collected in China in 2009, 2011, and 2019. Furthermore, the genome and molecular genetic evolution of TIBOVs isolated from different countries, periods, and hosts (mosquitoes, midges, and cattle) was systematically analyzed. The results revealed no molecular specificity among TIBOVs isolated from different countries, periods, and vectors. Meanwhile, the time-scaled phylogenetic analysis demonstrated that the most recent common ancestor (TMRCA) of TIBOV appeared approximately 797 years ago (95% HPD: 16-2347) and subsequently differentiated at least three times, resulting in three distinct genotypes. The evolutionary rate of TIBOVs was about 2.12 × 10^-3^ nucleotide substitutions per site per year (s/s/y) (95% HPD: 3.07 × 10^-5^, 9.63 × 10^-3^), which is similar to that of the bluetongue virus (BTV), also in the *Orbivirus* genus. Structural analyses of the viral proteins revealed that the three-dimensional structures of the outer capsid proteins of TIBOV and BTV were similar. These results suggest that TIBOV is a newly discovered and rapidly evolving virus transmitted by various blood-sucking insects. Given the potential public health burden of this virus and its high infectious rate in a wide range of animals, it is significant to strengthen research on the genetic variation of TIBOVs in blood-feeding insects and mammals in the natural environment and the infection status in animals.

## 1 Introduction

Orbivirus belongs to the genus *Orbivirus* within the family *Reoviridae*. The genus *Orbivirus* comprises 22 recognized viruses and 10 related but unclassified viruses identified by phylogenetic analysis based on the International Committee for the Taxonomy of Viruses (ICTV) guidelines (Matthijnssens et al., 2022). *Orbiviruses* are icosahedral and non-enveloped with a ten-segment (seg1~seg10) double-stranded RNA genome. The segments are of varying length and encode seven structural proteins (virus proteins, VP1-VP7) and four non-structural proteins (NS1-NS4) (Roy, 1996).

*Orbiviruses* are vector-borne pathogens with a wide range of vertebrate hosts. Various *Orbiviruses* are transmitted by blood-sucking insects such as mosquitoes, ticks, and lacewings, causing diseases in a wide range of vertebrates, including humans, domestic and wild ruminants, and rodents, which seriously threaten animal husbandry, and public health and safety (Matthijnssens et al., 2022). *Culicoides* (*Culicoides* spp.), one of the main orbivirus vectors, transmits a wide range of highly pathogenic and fatal diseases. For example, it transmits the bluetongue virus (BTV), which causes muscle necrosis, tissue edema, and several hemorrhagic diseases in cattle, sheep, and some African wildlife (Maclachlan, 2011). On the other hand, the African horse sickness virus (AHSV) causes acute or subacute cardiovascular and pulmonary diseases in horses and dogs, mainly manifested as bleeding, edema, and dyspnea. AHSV is highly infectious, with higher incidence and mortality rates. Based on this, AHSV adversely impacts the horse international trade and industry, given its significant economic losses (Zientara et al., 2015). Furthermore, the epizootic hemorrhagic disease virus (EHDV) causes several hemorrhagic diseases in ungulate animals, such as white-tailed deer, manifested as massive bleeding, dehydration, diarrhea, and death (Maclachlan et al., 2015). It was reported that BTV outbreaks caused economic losses of approximately $ 3 billion in 1996 worldwide. The total cost of preventing the incursion of BTV-8 into Scotland over the five-year period 2009-2013 is estimated to be about EUR14.1 billion (Saminathan et al., 2020). Severe epidemics of AHSV in the Middle East and south-west Asia between 1959 and 1963 resulted in the death of more than 300,000 horses (Dennis et al., 2019). In the fall of 2006, an outbreak of EHDV-7 in Israel caused economic losses ranging from US$1,591,000 to US$3,391,000, mainly related to reduced milk production (Jiménez-Cabello et al., 2023). In October 2015, an outbreak of EHDV-6 in Japanese cattle lasted for three months and affected 38 farms (Kamomae et al., 2018). It is clear that the orbivirus epidemic has had a severe impact on the global livestock economy.

Tibet orbivirus (TIBOV) was first isolated from *An. maculatus* mosquitoes collected in Xizang, southwestern China in 2009. Its original isolate, XZ0906, was classified as a novel virus species within the genus *orbivirus* (Li et al., 2014). Subsequently, a TIBOV strain, D181/2008, was isolated from *Culex fatigans* mosquitoes collected in Guangdong Province, southern China (Wu et al., 2020). In 2012 and 2013, two other TIBOV strains (DH13C120 and YN12246) were isolated from *Culicoides* samples collected in Yunnan Province, southwestern China (Lei et al., 2015; Wang et al., 2017). Since then, TIBOV has been isolated from various mosquito and midge species in southwestern China (Xing et al., 2017; Cao et al., 2019; Duan et al., 2021; Ren et al., 2021; Li et al., 2022; Duan et al., 2023). In addition, two TIBOV strains (KSB-8/C/09 and KSB-3/C/10) were isolated from midges in Japan Eastern Asia (Suda et al., 2021). In 2019, several TIBOV strains (GenBank ID: MH267259-MH267268) were also isolated from mosquitoes in Nepal South Asia. These reports indicate the rapid expansion of the geographic distribution of TIBOV and the range of its transmission vector, including mosquitoes and biting midges. Besides, animal experiments revealed that infection of lactating mice with TIBOV (strain DH13C120) causes serious illness and mortality (Wang et al., 2017). In addition, serological epidemiological surveys revealed positive TIBOV neutralizing antibodies in cattle and pigs (Lei et al., 2015; Wang et al., 2017), suggesting that TIBOVs are emerging viruses with significant potential animal disease burden.

However, although multiple TIBOV strains have been isolated from different regions, periods, blood-sucking vector species, and domestic cattle samples, the molecular genome structure, phylogenetic relationships, and evolutionary dynamics of TIBOV strains remain unclear. Therefore, this study first conducted complete genome sequencing of three TIBOV strains isolated from mosquito and midge specimens in China in 2009, 2011 and 2019. Subsequently, the genomic characteristics, molecular genetic evolution, and three-dimensional protein structure analysis of TIBOV strains isolated from different regions, vectors, and periods in Japan, Nepal, and China were investigated.

## 2 Materials and methods

### 2.1 Cells

*Aedes albopictus* C6/36 cells and BHK-21 (Baby hamster kidney) cells were kept in our laboratory. The BHK-21 cells were cultured in minimum essential medium (MEM) containing Eagle’s balanced salt solution supplemented with 10% fetal bovine serum (FBS, Invitrogen), 2 mM glutamine, 0.12% NaHCO_3_, 100U/mL penicillin and streptomycin, and incubated in a 5% CO_2_ incubator at 37°C. The C6/36 cells were cultured in a culture medium containing 45% RMPI 1640 and 45% DMEM (Invitrogen) supplemented with 10% FBS and 100 U/mL penicillin and streptomycin at 28°C in a 5% CO_2_ incubator (Li et al., 2014).

### 2.2 Viruses and their viral genomes sequencing

#### 2.2.1 Virus isolate strains

Three TIBOV isolates previously isolated from blood-sucking insects in China and preserved in our laboratory (Li et al., 2014; Cao et al., 2019; Wang et al., 2021), including isolates from *An. maculatus* mosquitoes (strain XZ0923) collected in Xizang, China, in the summer of 2009, from *Culex tritaeniorhynchus* mosquitoes collected in Hunan Province, China, in 2011 (strain HN11121), and from midges collected in Hainan Province, China, in summer 2019 (strain HNQZ1927) were used in this study.

#### 2.2.2 Cell infection of virus

Strains XZ0923 and HN11121 were inoculated into BHK-21 monolayers and continuously cultured until the viral suspensions showed the cytopathic effect (CPE). Next, the viral suspensions were dispensed and frozen at -80°C in a freezer (Li et al., 2014; Cao et al., 2019). Strain HNQZ1927 was inoculated into C6/36 cells. The viral suspension was harvested after the CPE was observed and frozen at -80°C (Wang et al., 2021).

#### 2.2.3 Viral genome sequencing

Total RNA was extracted from 140μl of BHK-21 or C6/36 cell supernatants using QIAamp Viral RNA Mini Kit (Qiagen) according to the manufacturer’s instructions. Next, the cDNA of the three TIBOV virus strains was synthesized using a Ready-To Go kit (GE Healthcare) using random hexanucleotide primers. The whole viral genome of the three TIBOV strains was then amplified using the 10 gene segment-specific amplification primers. Next, their nucleotide sequences in the gene amplification products were determined (Li et al., 2014). The viral genome sequences for the three virus isolates were deposited in the GenBank under the accession numbers (XZ0923: OR712119-OR712128(10 segments); HN11121: OR712129-OR712138(10 segments); HNQZ1927: OR712139-OR712146(8 segments, 2 segments were not sequenced successfully)).

### 2.3 Dataset construction

To understand the molecular biological characteristics, evolutionary relationship among the TIBOV strains, population origin in nature, and recent evolutionary dynamics of TIBOV, the whole genome of each TIBOV segment and nucleotide and amino acid sequences of the coding region were downloaded from the National Center for Biotechnology Information (NCBI), and together with the three strains in this study, a TIBOV genome sequence information dataset was constructed. All the viral gene sequences in the dataset included the background information on the virus isolation [isolation region, isolation date, host (vector)] and the viral genome nucleotide and amino acid sequences.

### 2.4 TIBOV sequence and phylogenetic analyses

The lengths of the nucleotides and amino acids in the coding sequences (CDS), initiation and termination codons, and 5’ and 3’ untranslated regions (UTRs) and their conserved sequences in each TIBOV segment were determined. Next, the genome structure of each TIBOV segment was visualized using the IBS software (Liu et al., 2015).

Subsequently, the existing CDS per viral gene segment in the dataset was aligned using Mafft (https://mafft.cbrc.jp/alignment/software) software. Furthermore, the MegAlign software in the DNAStar software package was used to analyze the similarity between nucleotide and amino acid sequences in the CDS of each TIBOV segment. The analysis results were visualized in heat maps constructed using the TBTools software (Chen et al., 2020). At the same time, the nucleotide base composition of the TIBOV 5’ UTR and 3’ UTR was calculated using the nucleotide composition function in Bioedit software, and the ggplot2 package in Rstudio was used to generate scatter plots showing the base (A, T, G and C) proportions in each segment per strain. Finally, the maximum likelihood tree was constructed using MEGA11 software (Tamura et al., 2021), with 1000 bootstrap replicates. The results were visualized on the ChiPlot website (Xie et al., 2023).

### 2.5 Time-scaled phylogenetic analyses of TIBOV

Most sequence information in the database constructed in this study was related to segment 10. Thus, phylogenetic analysis based on this segment could best demonstrate the evolutionary and transmission trends of virus strains from different geographies and vectors. Therefore, Bayesian analysis of the TIBOV genome segment 10 coding region sequences was performed using the BEAST v1.10.4 software package (Suchard et al., 2018). The Bayesian Markov chain Monte Carlo method (MCMC) was used to infer the maximum clade credibility (MCC), divergence time from the TMRCA, and the evolution rate. Recombination detection was performed using SplitsTreeCE (Huson, 1998). Phylogenetic signal detection was performed using iq-tree (Nguyen et al., 2015) in the phylosuite software (Zhang et al., 2020), and nucleotide substitution saturation was detected using Xia’s test (Xia, 2018) to determine the suitability of the dataset for phylogenetic analyses. In addition, the best-fit nucleotide substitution model was detected by ModelFinder (Kalyaanamoorthy et al., 2017) in phylosuite software. Furthermore, the relaxed clock model with different demographic models was tested, and the best models were selected based on the Bayes factor (BF) test using marginal likelihood values (2lnBF > 2). The chain length was set to 1 × 10^8^ to ensure effective mixing. The convergence of the parameters was detected using Tracer software (Rambaut et al., 2018) by monitoring the effective sample size > 200. The MCC tree was constructed using TreeAnnotator (http://beast.community/treeannotator) with 10% burn-in and visualized using the Figtree software (http://tree.bio.ed.ac.uk/software/figtree/). Meanwhile, the population dynamic history was inferred by Bayesian skyline reconstruction.

### 2.6 Three-dimensional structural analysis of the TIBOV VP5

The crystal structure of bluetongue virus VP5 protein (3j9e.1.A) was selected as the best template for homology modeling. SWISS-MODEL software (Waterhouse et al., 2018) and VMD (version 1.9.3) (Humphrey et al., 1996) and YASARA software (Land and Humble, 2018) were used to analyze and compare the protein tertiary structure and surface charge of VP5 of TIBOV and other orbiviruses.

## 3 Results

### 3.1 Establishment of TIBOV genome sequence dataset

#### 3.1.1 Background information on TIBOV

The isolation date, regions, vectors, and hosts of the 15 TIBOV strains obtained from the GenBank and the three viral sequences studied in this article are presented in **Table 1**. The 18 virus strains were isolated from blood-sucking vectors and mammals, including 7 from mosquitoes, 10 from midges, and 1 from cattle. The virus strains were isolated from China (Yunnan, Xizang, Hainan, Hunan, and Guangdong Provinces), Japan, and Nepal between 2007 and 2020. The GenBank data was included as of May 23, 2023.

**Table 1:** Background information on different TIBOV strains.

Strain	Host/Vector	Isolation area (date)	Genome segment (Genbank ID)										Reference
			Segment 1	Segment 2	Segment 3	Segment 4	Segment 5	Segment 6	Segment 7	Segment 8	Segment 9	Segment 10	
XZ0923*	*Anopheles maculatus*	Xizang, China (2009)	OR712119	OR712120	OR712121	OR712122	OR712123	OR712124	OR712125	OR712126	OR712127	OR712128	(Li et al., 2014)
HN11121*	*Culex tritaeniorhynchus*	Hunan, China (2011)	OR712129	OR712130	OR712131	OR712132	OR712133	OR712134	OR712135	OR712136	OR712137	OR712138	(Cao et al., 2019)
HNQZ1927*	midges	Hainan, China (2019)	OR712139	OR712140	OR712141	OR712142	OR712143	OR712144	–	OR712145	–	OR712146	(Wang et al., 2021)
SX-2017a	*Culex tritaeniorhynchus*	Yunnan, China (2007)	KX455487	KX455488	KX455489	KX455490	KX455491	KX455492	KX455493	KX455494	KX455495	KX455496	(Xing et al., 2017)
D181/2008	*Culex fatigans* mosquitoes	Guangdong, China (2008)	KR822286	KR822287	KR822288	KR822289	KR822290	KR822291	KR822292	KR822293	KR822294	KR822295	(Wu et al., 2020)
XZ0906	*Anopheles maculatus*	Xizang, China (2009)	KF746187	KF746188	KF746189	KF746190	KF746191	KF746192	KF746193	KF746194	KF746195	KF746196	(Li et al., 2014)
KSB-8/C/09	*Culicoides* sp.	Japan (2009)	LC567112	LC567113	LC567114	LC567115	LC567116	LC567117	LC567118	LC567119	LC5671120	LC567121	(Suda et al., 2021).
KSB-3/C/10	*Culicoides* sp.	Japan (2010)	LC567102	LC567103	LC567104	LC567105	LC567106	LC567107	LC567108	LC567109	LC567110	LC567111	(Suda et al., 2021).
HN11066	*Culex quinquefasciatus*	Hunan, China (2011)	–	–	–	–	–	–	–	–	–	MG731560	(Cao et al., 2019)
P110	*Anopheles annularis*	Nepal (2012)	MH267259	MH267260	MH267261	MH267262	MH267263	MH267264	MH267265	MH267266	MH267267	MH267268	**
YN12246	*Culicoides* spp.	Yunnan, China (2012)	KP099640Δ	–	KP099641	–	–	–	–	–	–	–	(Lei et al., 2015)
DH13C120	*Culicoides*	Yunnan, China (2013)	KU754026	KU754027	KU754028	KU754029	KU754030	KU754031	KU754032	KU754033	KU754034	KU754035	(Wang et al., 2017)
YN15-283-01	*Culicoides* spp.	Yunnan, China (2015)	MT793636	MT793637	MT793638	MT793639	MT793640	MT793641	MT793642	MT793643	MT793644	MT793645	(Ren et al., 2021)
V290/YNSZ	*Culicoides*	Yunnan, China (2019)	ON040940	ON040941	ON040942	ON040943	ON040944	ON040945	ON040946	ON040947	ON040948	ON040949	(Li et al., 2022)
V298/YNJH	sentinel cattle	Yunnan, China (2019)	ON040950	ON040951	ON040952	ON040953	ON040954	ON040955	ON040956	ON040957	ON040958	ON040959	(Li et al., 2022)
YNV/17-14	*Culicoides jacobsoni*	Yunnan, China (2020)	ON211599	ON211600	ON211601	ON211602	ON211603	ON211604	ON211605	ON211606	ON211607	ON211608	(Duan et al., 2023)
YNV/KM-1	*Culicoides*	Yunnan, China (2019)	ON211609	ON211610	ON211611	ON211612	ON211613	ON211614	ON211615	ON211616	ON211617	ON211618	(Duan et al., 2023)
KMV583	*Culicoides*	Yunnan, China (2020)	–	–	–	–	MW465962	–	–	–	MW465963	–	(Duan et al., 2021)

1) *The viral genome sequence information provided in this study.

2) **Only sequence information, no published information.

3) –: No sequence information.

#### 3.1.2 TIBOV genome dataset

Further analysis of the nucleotide/amino acid sequence information for each TIBOV gene segment revealed that some virus isolates had incomplete genomic sequence information (only a few nucleotide sequences in the CDS and UTRs), and some lacked the initiation and termination codon sequence information on the gene CDS. As a result, the sequences with incomplete genomic sequence information did not reflect the true characteristics of their viral genomic sequence. To this end, the genomic sequence of the TIBOV from the dataset (**Table 1**) were selected for the analysis of the molecular biological characteristics of the viral genome, including: 1) a complete nucleotide sequence with the CDS of the 1st to 10th gene segments; 2) The sequences on both sides of the CDS have clear initiation codon and termination codon information; 3) The 5 ’ and 3 ’ UTRs at both ends of the viral genome have complete conserved sequences (6 nucleotides). The genome sequences of the Tibet orbivirus isolates with the above sequence characteristics are shown in **Datasheet 1** and **Table 2**.

**Table 2:** UTRs (5’/3’) nucleotide sequences of the TIBOV genome.

Strain	5’ UTR Terminal sequence/length—3’UTR Terminal sequence/length									
	Seg1	Seg2	Seg3	Seg4	Seg5	Seg6	Seg7	Seg8	Seg9	Seg10
XZ0906△	GUAAAAUC/11 ACACUUAC/24	GUAAAAAC/13 AAACUUAC/34	GUAAAAUU/17 ACACUUAC/52	GUAAAAAC/8 ACACUUAC/38	GUAAAAAA/31 ACACUUAC/79	GUAAAAAA/26 AAACUUAC/29	GUAAAAAU/17 ACACUUAC/98	GUAAAAAA/20 AAACUUAC/42	GUAAAAAA/14 AAACUUAC/45	GUAAAAAA/21 CAACUUAC/106
SX-2017a	GUAAAAUC/11 ACACUUAC/24	GUAAAAAU/13 AAACUUAC/35	GUAAAAUU/17 AAACUUAC/52	GUAAAAAC/8 ACACUUAC/38	GUAAAAAA/31 ACACUUAC/79	GUAAAAAG/46 ACACUUAC/30	GUAAAAAU/17 ACACUUAC/98	GUAAAAAA/20 AAACUUAC/42	GUAAAAAA/14 AAACUUAC/45	GUAAAAAA/21 CAACUUAC/106
D181/2008	GUAAAAUC/11 ACACUUAC/24	GUAAAAAC/13 AAACUUAC/34	GUAAAAUU/17 –/44	GUAAAAAC/8 ACACUUAC/39	AAAAAAAU/31 ACACUUAC/79	GUAAAAAA/26 AAACUUAC/29	GUAAAAAU/17 ACACUUAC/98	GUAAAAAA/20 AAACUUAC/42	GUAAAAAA/14 AAACUUAC/45	GUAAAAAA/21 CAACUUAC/107
KSB-8/C/09	GUAAAAUC/11 ACACUUAC/24	GUAAAAUU/15 ACACUUAC/35	GUAAAAUU/17 ACACUUAC/52	GUAAAAAC/8 ACACUUAC/38	GUAAAAAA/31 ACACUUAC/78	GUAAAAAU/28 AAACUUAC/29	GUAAAAAU/17 ACACUUAC/98	GUAAAAAA/20 AAACUUAC/42	GUAAAAAA/14 AAACUUAC/45	GUAAAAAA/21 CAACUUAC/115
KSB-3/C/10	GUAAAAUC/11 ACACUUAC/24	GUAAAAAU/13 AAACUUAC/34	GUAAAAUU/17 ACACUUAC/52	GUAAAAAC/8 ACACUUAC/38	GUAAAAAA/31 ACACUUAC/78	GUAAAAAA/29 ACACUUAC/28	GUAAAAAU/17 ACACUUAC/98	GUAAAAAA/20 AAACUUAC/42	GUAAAAAA/14 AAACUUAC/45	GUAAAAAA/21 CAACUUAC/106
P110	GUAAAAUC/11 ACACUUAC/9	GUAAAAAU/13 ACACUUAC/35	GUAAAAUU/17 ACACUUAC/52	GUAAAAAC/8 –/13	GUAAAAAA/31 ACACUUAC/77	GUAAAAAA/29 AAACUUAC/32	GUAAAAAU/17 ACACUUAC/98	GUAAAAAA/20 AAACUUAC/42	GUAAAAAA/14 AAACUUAC/45	GUAAAAAA/21 CAACUUAC/115
DH13C120△	GUAAAAUCA/11 AUACACUUAC/24	GUAAAAAUC/13 UUAAACUUAC/35	GUAAAAUUU/17 AUACACUUAC/52	GUAAAAACA/8 UUACACUUAC/38	GUAAAAAAG/31 UUACACUUAC/79	GUAAAAAGA/31 UUUCACUUAC/30	GUAAAAAUU/17 UUACACUUAC/98	GUAAAAAAU/20 UUAAACUUAC/42	GUAAAAAAU/14 UAAAACUUAC/45	GUAAAAAAG/21 CCCAACUUAC/106
YN15-283-01	GUAAAAUC/11 ACACUUAC/24	GUAAAAAC/13 AAACUUAC/35	GUAAAAUU/17 AAACUUAC/52	GUAAAAAC/8 ACACUUAC/38	GUAAAAAA/31 ACACUUAC/79	GUAAAAAA/29 AAACUUAC/30	GUAAAAAU/17 ACACUUAC/98	GUAAAAAA/20 AAACUUAC/42	GUAAAAAA/14 AAACUUAC/45	GUAAAAAA/21 CAACUUAC/107
V290/YNSZ	GUAAAAUC/11 ACACUUAC/24	GUAAAAAU/13 AAACUUAC/36	GUAAAAUU/17 AAACUUAC/52	GUAAAAAC/8 ACACUUAC/38	GUAAAAAA/31 ACACUUAC/78	GUAAAAAA/29 AAACUUAC/30	GUAAAAAU/17 ACACUUAC/97	GUAAAAAA/20 AAACUUAC/42	GUAAAAAA/14 AAACUUAC/45	GUAAAAAA/21 CAACUUAC/115
V298/YNJH	GUAAAAUC/11 ACACUUAC/24	GUAAAAAU/13 AAACUUAC/35	GUAAAAUU/17 AAACUUAC/52	GUAAAAAC/8 ACACUUAC/38	GUAAAAAA/31 ACACUUAC/86	GUAAAAAA/26 AAACUUAC/29	GUAAAAAU/17 ACACUUAC/98	GUAAAAAA/20 AAACUUAC/42	GUAAAAAA/14 AAACUUAC/45	GUAAAAAA/21 CAACUUAC/115
YNV/17-14	GUAAAAUC/11 ACACUUAC/24	GUAAAAAU/13 ACACUUAC/38	GUAAAAUU/17 ACACUUAC/52	GUAAAAAC/8 ACACUUAC/38	GUAAAAAA/31 ACACUUAC/78	GUAAAAAA/28 GCACUUAC/28	GUAAAAAU/17 ACACUUAC/98	GUAAAAAA/20 AAACUUAC/42	GUAAAAAA/14 AAACUUAC/45	GUAAAAAA/21 CAACUUAC/115
YNV/KM-1	GUAAAAUC/11 ACACUUAC/24	GUAAAAAU/13 AAACUUAC/36	GUAAAAUU/17 AAACUUAC/52	GUAAAAAC/8 ACACUUAC/38	GUAAAAAA/31 ACACUUAC/78	GUAAAAAA/26 AAACUUAC/29	GUAAAAAU/17 ACACUUAC/98	GUAAAAAA/20 AAACUUAC/42	GUAAAAAA/14 AAACUUAC/45	GUAAAAAA/21 CGACUUAC/115

1) △: The strains XZ0906 and DH13C120 gene sequences were previously reported [6] and [8], respectively.

2) –: The 3’ UTRs conserved sequence of strain D181/2008 segment 3 and strain P110 segment 4 were deleted, possibly related to sequencing errors.

3) The length of 5’ UTRs of the TIBOV genome was between 8 (XZ0906 strain) and 31 (XZ0906 strain) nucleotides, with 6 nucleotide conserved sequences. The length of 3’ UTRs was between 9 (P110 strain) and 107 (D181/2008 strain) nucleotides, with 6-nucleotide conserved sequences.

4) The 5’ -end conserved sequence of the D181/2008 segment 2 was inconsistent, possibly related to sequencing error.

Molecular biological characteristic analysis revealed that TIBOV had ten segments (1-10) and was a double-stranded RNA virus. The initiation codon at the coding region across the ten segments in all TIBOV strains was ATG, and the termination codons were TAA, TGA, or TAG (**Datasheet 1**).

### 3.2 TIBOV genome

#### 3.2.1 TIBOV genome structure

**Figure 1:**
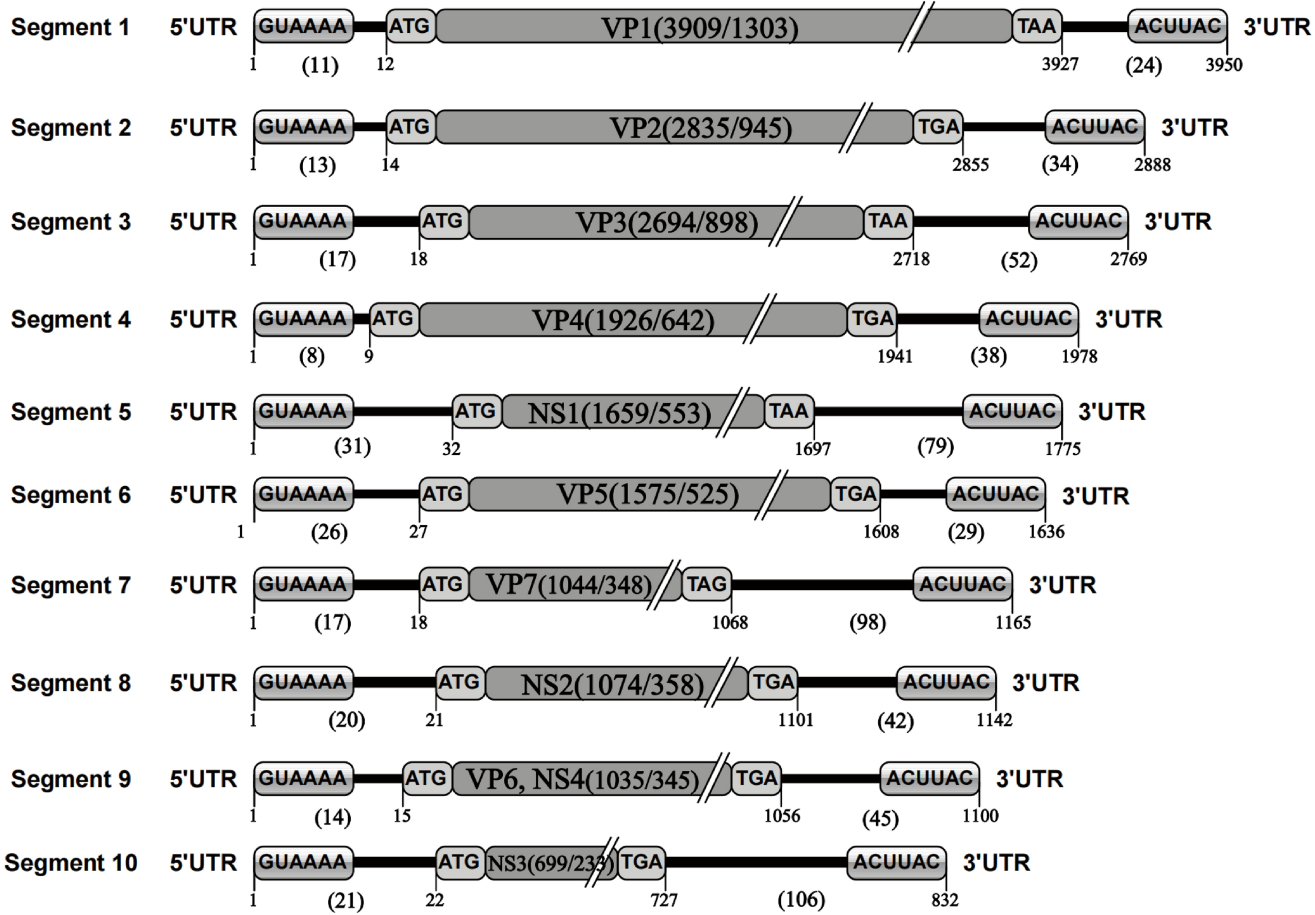
TIBOV genome structure. 1 represents the first nucleotide at the 5’ end of the genome; 11 represents the length of the nucleotide sequence at the 5’ UTR of the gene segment; 12 represents the initial nucleotide site of the genome CDS; GUAAAA is the 5’ conserved sequence; ATG is the CDS initiation codon; VP1 (3909/1303) is the nucleotide/amino acid length of the genome coding region (VP1) excluding codons; TAA is the CDS termination codon; 3927 is the UTR starting site; 24 is the 3’ UTR nucleotide sequence length in the genome; ACUUAC is a conserved sequence at the end of the 3’ UTR; 3950 is the total length of the nucleotide sequence of the gene segment. The genetic elements of segments 2 to 10 were the same. Given the TIBOV strains analyzed in this study came from different laboratories in different countries, the sequencing accuracy could be affected by various factors such as the sequencing institutions, sequencing methods, instrumentation, and operating methods; thus, errors (differences in the lengths of the genomic nucleotide sequences) would inevitably occur. However, given the length of most TIBOV genomic sequences across the various stains is the same as the original TIBOV strain (strain XZ0906) sequence, the determination of sequence lengths and codons in the genome structure schematic in this study is based on two principles: 1) conformity principle, which is based on the sequence length of most strains and ignores the information of individual sequences; 2) Where there were sequence differences, the XZ0906 strain sequence was used as the standard, since the XZ0906 virus strain was first sequenced using a large-scale deep sequencing method (454 sequencing) to obtain the TIBOV genome 10-segment nucleotide sequence. Before sequencing the genome by deep sequencing, a set of specific amplification primers for the 10 gene segments were designed and synthesized. The XZ0906 virus strain cDNA was used as a template for gene amplification (PCR) of 10 gene segments. Subsequently, the gene amplification products were sequenced and spliced to obtain the whole genome sequence information. In addition, the UTR sequence of the XZ0906 virus genome was obtained using the 5’ and 3’ RACE systems amplification method.

The nucleotide sequence analysis of the TIBOV genome revealed that the TIBOV genome was a 10-segment double-stranded RNA virus, and each gene segment was composed of 5’ UTR, CDS, and 3’ UTR. Furthermore, the nucleotide sequence length of each virus genome segment was different. The length was sequentially decreased from the segment 1 genome (the largest with 3950 nucleotides) to segment 10, which was only 832 nucleotides (**Figure 1**). The 10-segment genomes encoded four non-structural proteins NS1-NS4 (corresponding to segments 5, 8, 9 and 10, respectively) and seven structural proteins VP1-VP7 (corresponding to segments 1, 2, 3, 4, 6, 7, and 9, respectively).

The nucleotide length of the 5’ UTR ranged from 8 nucleotides (segment 4) to 31 nucleotides (segment 5). There was also a conserved sequence (GUAAAA-) of 6 nucleotides in the 5’ UTR of each segment at the beginning of the 5’ end of the TIBOV genome sequence (**Table 2**; **Figure 1**). Moreover, the CDS nucleotide sequence length (the initiation codon is ATG) of each TIBOV strain was different. The CDS nucleotide length of segment 1 was 3909 nucleotides and was sequentially decreased, with segment 10 having 717 -726 nucleotides. (**Datasheet 1**; **Figure 1**). The CDS termination codons were unique in six of the 10 gene segments (TAA, TAG, TGA) and variable in the remaining four segments (TAA, TGA, TAG) (**Datasheet 1**; **Figure 1**). Besides, there was no correlation between the termination codons and the host (mosquito, midge or mammal), geographical region and the virus isolation date. The length of the 3’ UTRs ranged from 9 nucleotides (segment 1) to 116 nucleotides (segment 10). In addition, there was a conserved sequence (–ACUUAC) of 6 nucleotides in the 3’ UTR of each segment at the end of the 3’ UTR of the TIBOV genome sequence (**Table 2**, **Figure 1**).

#### 3.2.2 Nucleotide/amino acid sequence similarity of the TIBOV coding region

**Figure 2:**
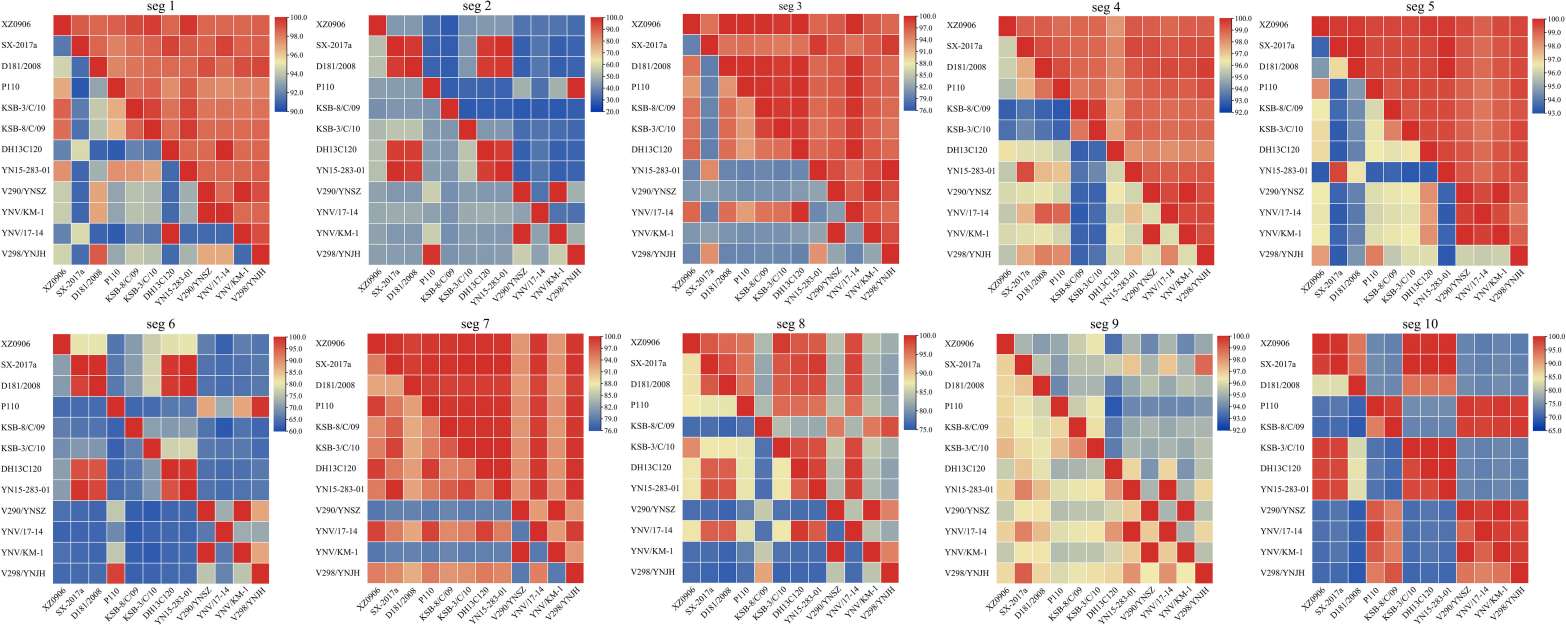
Nucleotide and amino acid similarity between the 10 TIBOV gene segments. The nucleotide and amino acid similarities among the 10 gene segments (seg 1 ~ seg 10) of each strain are shown in the 10 sub-graphs. The diagonal lines connected to the upper left and lower right corners of each segment indicate that the nucleotide (amino acid) similarity between the same strains is 100%. The amino acid similarity above the diagonal line and the nucleotide similarity below the diagonal line. The horizontal and vertical axes represent the name of the strain, and the scale represents the similarity (%).

Analysis of the nucleotide and amino acid similarity across the TIBOV gene segments revealed that the nucleotide and amino acid sequences were different across the various gene segments, and the difference was most obvious in segments 2 and 6. For example, the nucleotide and amino acid similarity of segment 2 in KSB-8/C/09 and V290/YNSZ strains was only 41.6% and 27.8%, respectively. In segment 6, the nucleotide and amino acid similarities were only 61.6% and 61.3%, respectively, in KSB-8/C/09 and YNV/17-14 strains. However, the nucleotide and amino acid similarities between the other gene segments were more than 70% (**Figure 2**).

#### 3.2.3 Nucleotide sequence characteristics of the untranslated region of the TIBOV genome

**Figure 3:**
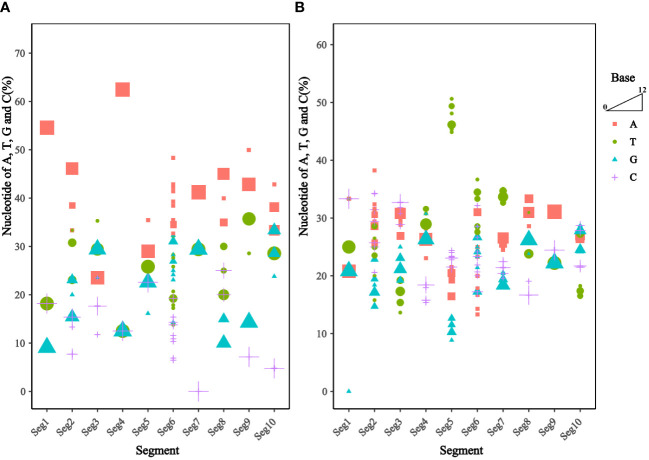
Frequency of nucleotide sequences (A, T, G, and C) in the UTR of the different TIBOV strains genome. **(A)** Nucleotide content in 5’ UTR. **(B)** Nucleotide content in 3’ UTR. Horizontal coordinates are TIBOV virulent strain segments, and vertical coordinates are a percentage of nucleotide content. Red squares indicate adenine (A), green circles indicate thymine (T), blue triangles indicate guanine (G), and purple crosses indicate cytosine (C). The trapezoid indicates that the larger the shape, the more the number of strains.

Analysis of the base content of the TIBOV 5’ UTR and 3’ UTR (**Figure 3**) revealed that the 5’ UTR base composition was similar in each segment. In addition, the base content in the segment 1 and segment 7 were the same across the various strains but quite different in the segment 2 and segment 6. In segment 6, the base content between the TIBOV strains was different, except for the base content between the XZ0906 and V298/YNSZ strains. Overall, A>T>G>C in 5’ UTR with A, T, G, and C contents ranging between 23.53 ~ 62.50%, 12.50 ~ 35.71%, 9.09 ~ 33.33%, and 0 ~ 25.00%, respectively.

On the contrary, the 3’ UTR base composition varied widely in each segment. Except for segment 9, where the base composition was identical in all the TIBOV strains, the base composition in the rest of the segments was different, with the largest differences in segments 2, 5, and 6. For example, the base content in segment 2 varied among the TIBOV strains, except for the V290/YNSZ and YNV/KM-1 strains, which had the same base content. The A, T, G, and C contents in 3’ UTR ranged between 13.33 ~ 38.24%, 13.64 ~ 50.63%, 0 ~ 30.77%, and 15.38 ~ 34.29%, respectively.

### 3.3 Phylogenetic analysis of the TIBOV genome

**Figure 4:**
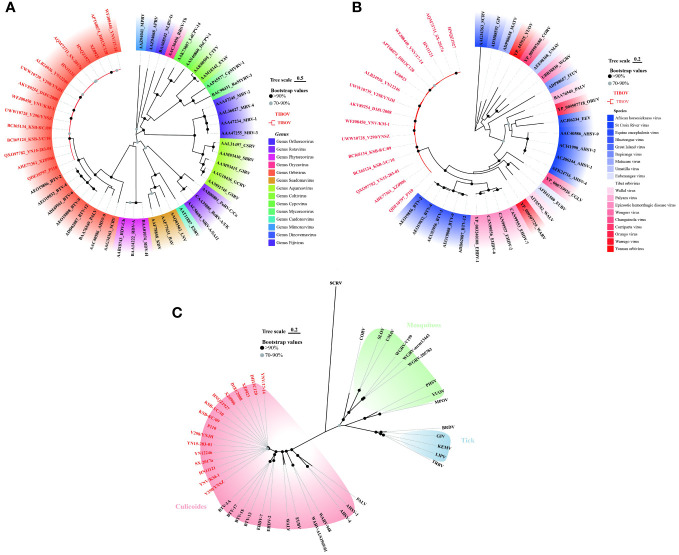
Phylogenetic analysis of VP1 amino acid sequences from **(A)** Reoviridae and **(B)** Orbivirus genus. **(C)** Phylogenetic analysis of T2 amino acid sequences from Orbivirus strains. The tree scale indicates the number of amino acid substitutions per site. Bootstrap values are indicated by dots at the end of the branch, black dots indicate values above 90%, and values between 70% and 90% are indicated by grey dots. The same color shading in panel **(A)** indicates a virus of the same genus; in panel **(B)** same color shading indicates a virus of the same species; and in panel **(C)**, the same color shading indicates transmission by the same vector in an evolutionary relationship. TIBOV strain names are highlighted in red. The best-fit evolutionary models were calculated as LG+G (4A), LG+G (4B), and LG+G+F (4C) using the find best models function in MEGA11 software, respectively.

To clarify the molecular genetic taxonomic classification of TIBOV in the family *Reoviridae*, the amino acid sequences of 37 VP1 proteins (**Datasheet 2**) distributed across 14 genera within the family *Reoviridae* were obtained from the GenBank. Subsequently, the 37 VPI proteins and those of TIBOV (including the three strains provided in this study) were used to construct a maximum likelihood phylogenetic tree. Phylogenetic analysis revealed that the *Reoviridae* viruses clustered into 14 evolutionary branches, with TIBOV clustering within the branch containing VPI proteins from the genus *Orbivirus* (**Figure 4A**). To further establish the taxonomic classification of TIBOV, VP1 amino acid sequences from 28 known *Orbivirus* strains and TIBOV VP1 proteins (including the three strains provided in this study) (**Datasheet 2**) were used to construct a phylogenetic maximum likelihood tree specific to the *Orbivirus* genus. The phylogenetic analysis of the VP1 amino acid sequences from the *Orbivirus* strains, including TIBOV, revealed that all the TIBOV isolates clustered into a separate evolutionary branch independent of any known *Orbivirus* species (**Figure 4B**).

The T2 protein constitutes the inner capsid of the viral particle, highly conserved in the genus *Orbivirus*, so the analysis of the amino acid sequence of the T2 protein is an important basis for identifying species in the genus *Orbivirus* (Matthijnssens et al., 2022). T2 amino acid sequences from 28 known *Orbivirus* strains and the TIBOV equivalent region were selected to construct a maximum likelihood phylogenetic tree. Further phylogenetic analysis of the T2 amino acid sequences revealed that the TIBOV clustered into a separate evolutionary branch and did not belong to any other known species in the genus *Orbivirus* (**Figure 4C**).

These results reveal that TIBOV belongs to the genus *Orbivirus* of the family *Reoviridae* and represents a novel species within the genus *Orbivirus.*

### 3.4 Spatial and temporal dynamics analysis of TIBOV

#### 3.4.1 Time scale phylogenetic analysis of TIBOV

**Figure 5:**
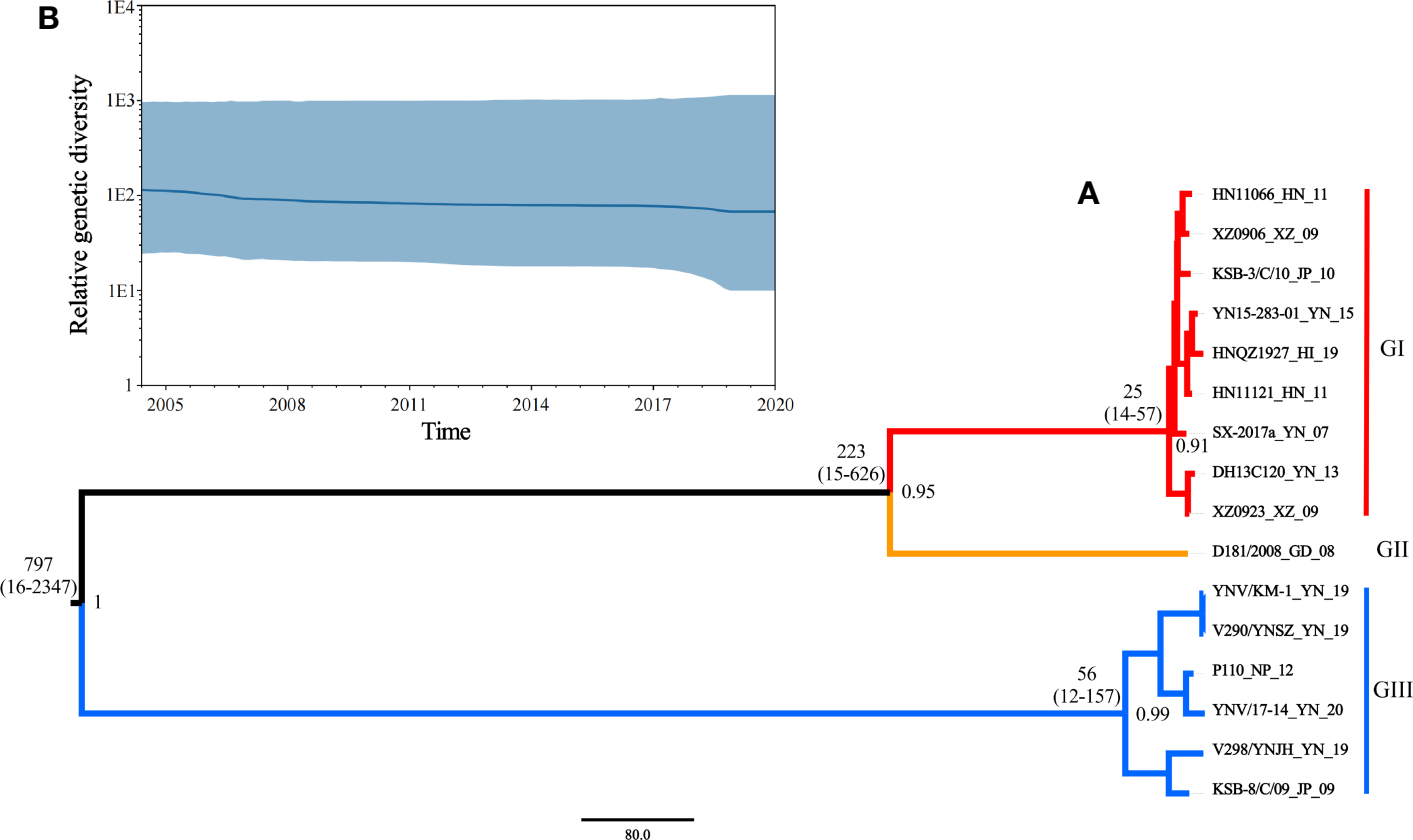
**(A)** Maximum clade credibility tree for coding sequences of segment 10 of TIBOV. GI (red), GII (orange), and GIII (blue) indicate the three distinct sublineages. The estimated TMRCAs of these lineages (with 95% HPD values in parentheses) were GI: 25 (14-57) and GIII: 56 (12-157), respectively. The posterior probability value of each branch is displayed on the right of the node. **(B)** Bayesian skyline plots of the TIBOV population diversity against time. Highlighted areas correspond to 95% HPD intervals. The horizontal and vertical axes represent time and genetic diversity, respectively.

Based on the phi test, there was no statistically significant evidence of recombination (p = 0.9433). Phylogenetic signal detection on segment 10 CDS region revealed the star-like (n) value was 2.5% < n < 30%, implying that the data set was well analyzed. The nucleotide substitution saturation was I_SS_ < I_SS.C_, with insignificant differences, implying the nucleotide sequence substitution was not saturated. These results suggest that the data set was suitable for phylogenetic analysis. The base substitution model detected by ModelFinder in phylosuite software was HKY + F + G4. According to the Bayesian factor and the 95% HPD interval, the Bayesian skyline model with a relaxed (uncorrelated lognormal) molecular clock model was selected as the best-fitting model. Based on Bayesian MCMC analysis, the MCC tree (**Figure 5A**) was established. The posterior probability values of all branch nodes were greater than 0.9, indicating its robustness. The results revealed that the TIBOV TMRCA appeared about 797 years ago (95% HPD: 16-2347), and three different lineages evolved during evolution, resulting in three distinct genotypes. Viral populations of genotype I appeared approximately 25 years ago (95% HPD: 14-57) and genotype III approximately 56 years ago (95% HPD:12-157). Genotype II contained only one strain, so TMRCA was not estimated.

The results also revealed that the TIBOV was divided into three genotypes. Genotype I included nine strains, including HN11066, XZ0906, KSB-3/C/10, YN15-283-01, HNQZ1927, HN11121, SX-2017, DH13C120 and XZ0923. Genotype II contained only one strain (D181/2008). Genotype III included YNV/KM-1, V290/YNSZ, P110, YNV/17-14, V298/YNJH and KSB-8/C/09 strains. The strains isolated from Japan were in genotypes I and III, the Chinese isolates were distributed in all three genotypes, and the Nepal isolates were in genotype III.

#### 3.4.2 Genome evolution rate and population dynamics of TIBOV

According to the Bayesian MCMC algorithm, the mean nucleotide substitution rate for the TIBOV segment 10 sequence was estimated as 2.12 × 10^-3^ nucleotide substitutions per site per year (s/s/y) (95% HPD values, 3.07 × 10 ^− 5^, 9.63 × 10 ^− 3^).

The skyline plot of the TIBOV population dynamics is shown in **Figure 5B**. The TIBOV population dynamic was relatively stable during evolution. However, from 2007 to 2009, the TIBOV population diversity decreased slightly. After 2011, the population diversity remained relatively stable until a slight decline was recorded between 2018 and 2020.

### 3.5 Comparative analysis of the VP5 proteins three-dimensional structure between TIBOV and BTV

**Figure 6:**
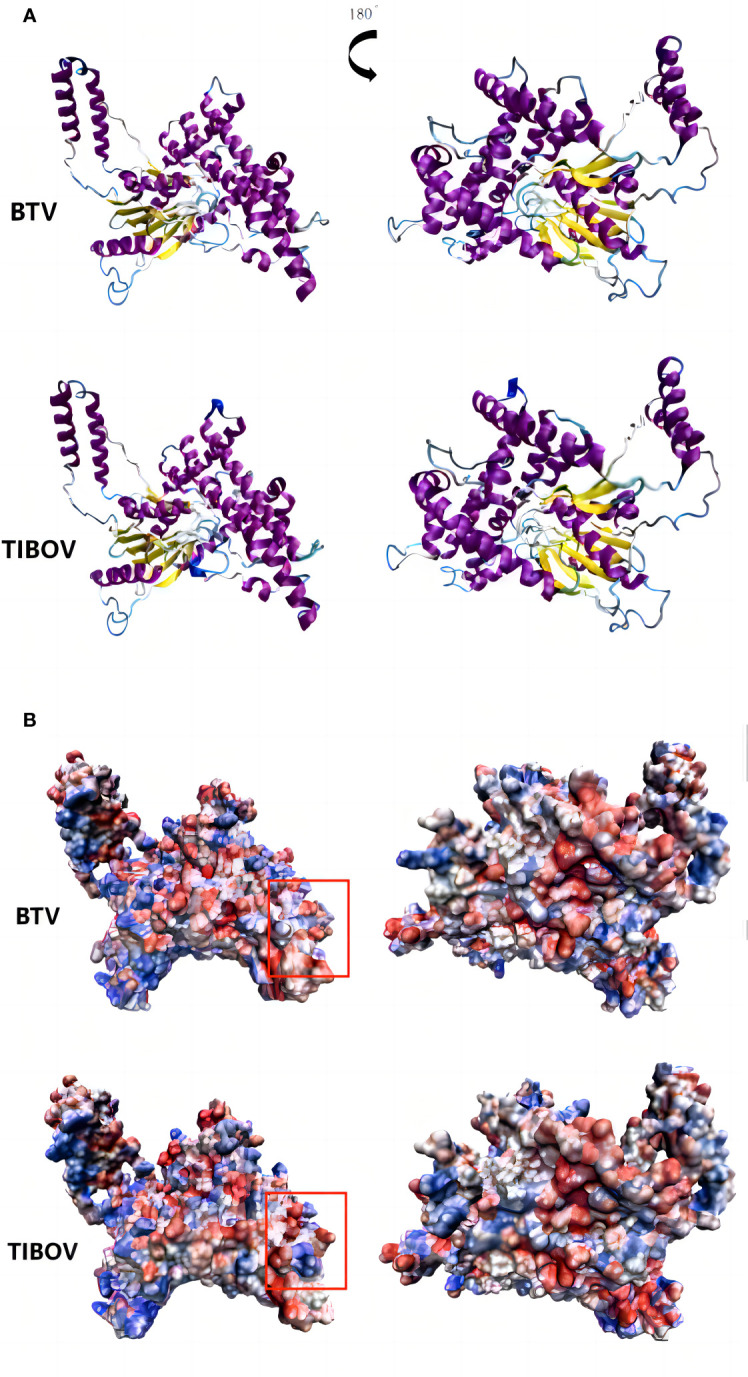
Three-dimensional structure and surface charge model of VP5 of bluetongue virus and Tibet orbivirus. panel **(A, B)** are the three-dimensional structure of the bluetongue virus and the Tibet orbivirus and their surface charge model positive maps and their rotated 180° planes, respectively. In panel **(B)**, blue represents positive charge and red represents negative charge.

The TIBOV segment 6 gene encodes the structural protein VP5, a viral capsid protein. VP5 acts as a membrane fusion protein associated with virus serotyping. The three-dimensional structures of the VP5 proteins encoded by TIBOV (XZ0906 strain) and BTV (Genbank ID: YP_052955) are presented in **Figure 6**.

The surface charge distribution of the TIBOV VP5 protein was similar to that of the BTV VP5 protein, with minor differences. In the annotated region of the orthographic view, the TIBOV VP5 protein was positively charged in the middle and negatively charged on both sides. On the other hand, the BTV VP5 protein exhibited a negative charge in the middle and positive charges on both sides.

## 4 Discussion

### 4.1 The genome and spread of TIBOV

TIBOV molecular genetic evolution analysis revealed that the viruses isolated from mosquitoes, midges, and mammalian (cattle) specimens were dispersed in different evolutionary branches, with no vector or host-animal-specific clustering. Viral isolates from mosquito and midge were clustered in the evolutionary branches with genotypes I and III viruses. The genotype III evolutionary branch hosts included Anopheles mosquitoes, midges (given midges have not been taxonomically characterized, the 10 viral strains may have been isolated from different blood-sucking midges), and mammals (cattle). Furthermore, the TIBOV in genotype III was isolated from different countries, including China, Japan, and Nepal. TIBOV is highly host (vector and mammal) adapted, and there is no barrier to cross-species transmission between vectors or between vectors and host animals (cattle). In this study, the viral phylogenetic analysis revealed that the viruses isolated from Nepal in South Asia and Japan in East Asia are in the same evolutionary branch (genotype III) as those isolated from China, implying that the TIBOV genome is not geographically clustered. This characteristic of TIBOV is similar to that of BTV, another double-stranded RNA virus with a 10-gene segment, whose genome also lacks the vector and host aggregation characteristics (Wilson et al., 2000; Biswas et al., 2021). This is also one of the major reasons why BTVs are widely distributed in Africa (Coetzee et al., 2012), Europe (Zientara and Sánchez-Vizcaíno, 2013) and Asia (Yang et al., 2021). The molecular genetic evolution analysis of the mosquito-borne Japanese encephalitis virus widespread in Asia also revealed that the Japanese encephalitis viruses isolated from different mosquito vectors (Culex, Anopheles, and Aedes), pigs, and patients are distributed in the same evolutionary branches (Pan et al., 2011). However, the TIBOV genomes isolated from different vectors and mammals lack the relative aggregation of species, which may be the molecular basis for the cross-species transmission and geographical expansion of TIBOV.

### 4.2 TIBOV genome and geographical distribution

TIBOV was first isolated from mosquitoes in southwestern China in 2009. Since then, the virus has been isolated in several other provinces in China, Japan (Suda et al., 2021), and Nepal (Accession Number: MH267259-MH267268), suggesting that its geographic distribution is expanding rapidly.

Time-scale phylogenetic analyses in this study revealed that TMRCA of TIBOV appeared about 797 years ago (95% HPD: 16-2347) and subsequently evolved into three distinct lineages, giving rise to three distinct genotypes (GI,GII and GIII). Genotype I appeared about 25 years ago (95% HPD: 14-57) and genotype III about 56 years ago (95% HPD: 12-157), suggesting that TIBOV is a relatively young virus population in the viruses evolution; thus, it is an emerging virus. Besides, the average nucleotide substitution rate of TIBOV was 2.12 × 10^-3^ nucleotide substitutions per site per year (95% HPD values, 3.07 × 10^-5^, 9.63 × 10^-3^). The nucleotide substitution rates of TIBOV were similar to that of BTV, another 10-segmented double-stranded RNA viruses highly pathogenic to a wide range of animals. TIBOV evolution rate is similar to that of BTV (1.330 × 10^-4^, (6.101 × 10^-5^-2.294 × 10^-4^, 95% HPD)) (Biswas et al., 2021). The absence of a vector and host animal barrier for TIBOV is another reason this virus rapidly achieved geographically long-distance expansion in a short period.

TIBOV has been isolated from *Cx. tritaeniorhynchus* and *Culex. quinquefasciatus* (Xing et al., 2017; Cao et al., 2019; Wu et al., 2020), which carry and transmit more than 10 arboviruses (Liang et al., 2018). In particular, they are the vectors of the Japanese encephalitis virus, which is widely prevalent in Asia (Zheng et al., 2012). *Cx. quinquefasciatus* is distributed in almost all continents, including Asia, Africa, and North and South America (Farajollahi et al., 2011). On the other hand, *Cx. tritaeniorhynchus* is mainly distributed in Asia (Longbottom et al., 2017). Given the wide global distribution of *Cx. tritaeniorhynchus* and *Cx. quinquefasciatus*, the geographical ranges of the viruses they carry and transmit will change in response to rising temperatures, particularly with climate warming (Ciota and Keyel, 2019), allowing TIBOV to move to higher latitudes with vector mosquitoes. Currently, TIBOVs are only prevalent in South and East Asia. However, in the future, TIBOVs will likely spread with mosquito vectors to higher latitudes in Eurasia due to climate change.

### 4.3 TIBOV animal infection

TIBOV animal infection experiments revealed that intracerebral injection of TIBOV (DH13C120 strain) in suckling mice induced tremors and neck stiffness at 48 h post-infection, and all infected mice died within 72 h post-infection (Wang et al., 2017). In suckling BALB/c mice, intracranial injection of TIBOV (V290/YNSZ strain) decreased the feed intake activity and induced hind limbs paralysis. The mice started dying 3 days post-infection (dpi), and all animals died at 6 dpi (Li et al., 2022), implying that TIBOV is pathogenic and lethal to animals.

Animal seroepidemiological surveys have also revealed TIBOV (DH13C120 strain) neutralizing antibody positivity in cattle (44% (22/50)), buffalo (20% (12/60)) and goats (4% (1/25)) in Yunnan Province, China (Wang et al., 2017). Besides, the TIBOV neutralizing antibody positivity was 4-fold or higher in acute and convalescent sera collected from locally affected livestock, implying that TIBOV may also cause infection and morbidity in animals such as goats and cattle (Wang et al., 2017). Detection of the IgG antibody levels of TIBOV (YN12246 strain) in swine serum samples by the indirect immunofluorescence assay revealed that the positive rate of antibody was 14.0% (8/57) (Lei et al., 2015). Furthermore, TIBOV infection analysis by PCR on 340 blood samples from cattle, goats, and swine in Yunnan Province of Southwest China showed that 36 blood samples (10.6%), including 16.4% (18/110) for cattle, 10.8% (13/120) for goats, and 5.5% (6/110) for swine) were positive for TIBOV. The Ct values ranged from 30.8 to 36.4 (Li et al., 2022). These reports indicate that TIBOV is not only infectious to cattle, goats, swine, and other domestic animals but also has high viremia characteristics, similar to BTV (Dal Pozzo et al., 2009).

BTV is mainly transmitted by the bite of blood-sucking midges of the genus *Culicoides* and family *Ceratopogonidae*. It infects domestic animals such as sheep, goats, cattle, and wild animals (Saminathan et al., 2020). BTV-positive livestock has been reported in 29 provinces in Northeast, North, Northwest, East, Central and South China (Gong et al., 2020). Most TIBOV strains have been isolated from midge specimens collected in various parts of China, suggesting that midges may also be the TIBOV vector. Moreover, antibodies against TIBOV have been detected in several even-toed ungulate animals in China, such as cattle and sheep (Wang et al., 2017; Li et al., 2022). These facts suggest that TIBOV and BTV, widely prevalent in China, are highly overlapping regarding vector and host. Based on this, it is likely that TIBOV is similar to BTV, widely prevalent in mainland China and infecting large animals. Besides, the findings in this study revealed that the three-dimensional structure of TIBOV and BTV protein is very similar, with slight changes in the surface charge distribution. In addition, the genome evolution rate of TIBOV is similar to that of BTV. Based on this, TIBOV may further spread within the original distribution range and evolve into new virus strains that can cause cattle, sheep, and other large livestock diseases. Therefore, it is significant to strengthen the research on TIBOV genomic variation in blood-sucking insects and mammals, its infection in domestic and wild animals, and its public health burden.

### 4.4 Limitations of this study

This study revealed the systematic, molecular, and biological characteristics of the TIBOV genome sequences isolated to date, including the TIBOV genome structure, homology, and molecular genetic evolution. The major shortcoming of this study is the limited number of strains studied, especially the few isolated at different times and in different geographical areas. For example, one virus (Genbank ID: MH267259-MH267268) was isolated from Nepal and cattle among the animal specimens (Li et al., 2022). Therefore, the geographical distribution of TIBOVs and the identification of host animals need to be analyzed with more strains for more accurate results. Besides, given the limited number of TIBOV strains in this study, the TMRCA and migration route data obtained in this study must be verified by gene sequences from more strains.

## Data availability statement

The datasets presented in this study can be found in online repositories. The names of the repository and accession numbers can be found in the article and **Supplementary Material**.

## Author contributions

TG: Writing – original draft, Writing – review & editing. ML: Writing – original draft, Writing – review & editing. HL: Writing – original draft, Writing – review & editing. SF: Writing – review & editing. HW: Writing – review & editing. GL: Writing – original draft, Writing – review & editing.
