# Supplementary material for: Genome and evolution of Tibet orbivirus, TIBOV (genus Orbivirus, family Reoviridae)
Source: Front Cell Infect Microbiol. 2024 Mar 5;14:1327780. doi: 10.3389/fcimb.2024.1327780 (PMC10950067; doi:10.3389/fcimb.2024.1327780)
Supplement: Supplementary file 1 [file DataSheet_1.pdf]

Data Sheet 1. Nucleotide/amino acid sequence of the TIBOV gene CDS

Strain	Seg1			Seg2			Seg3			Seg4			Seg5			Seg6			Seg7			Seg8			Seg9			Seg10		
	IC	Length	TC	IC	Length	TC	IC	Length	TC	IC	Length	TC	IC	Length	TC	IC	Length	TC	IC	Length	TC	IC	Length	TC	IC	Length	TC	IC	Length	TC
XZ0906	ATG	3909/1303	TAA	ATG	2835/945	TGA	ATG	2694/898	TAA	ATG	1926/642	TGA	ATG	1659/553	TAA	ATG	1575/525	TGA	ATG	1044/348	TAG	ATG	1074/358	TGA	ATG	1035/345	TGA	ATG	699/233	TGA
SX-2017a	ATG	3909/1303	TAA	ATG	2847/949	TAG	ATG	2694/898	TAA	ATG	1926/642	TGA	ATG	1659/553	TAA	ATG	1575/525	TGA	ATG	1044/348	TAG	ATG	1074/358	TGA	ATG	1038/346	TAA	ATG	699/233	TGA
D181/2008	ATG	3909/1303	TAA	ATG	2847/949	TAG	ATG	2694/898	TAA	ATG	1926/642	TGA	ATG	1659/553	TAA	ATG	1575/525	TGA	ATG	1044/348	TAG	ATG	1083/361	TGA	ATG	1035/345	TAA	ATG	699/233	TGA
KSB-8/C/09	ATG	3909/1303	TAA	ATG	2715/905	TAA	ATG	2694/898	TAA	ATG	1926/642	TGA	ATG	1659/553	TAA	ATG	1575/525	TGA	ATG	1044/348	TAG	ATG	1074/358	TGA	ATG	1035/345	TGA	ATG	690/230	TAA
KSB-3/C/10	ATG	3909/1303	TAA	ATG	2835/945	TAA	ATG	2694/898	TAA	ATG	1926/642	TGA	ATG	1659/553	TAA	ATG	1575/525	TGA	ATG	1044/348	TAG	ATG	1074/358	TGA	ATG	1035/345	TGA	ATG	699/233	TGA
P110	ATG	3924/1308	TGA	ATG	2760/920	TAG	ATG	2694/898	TAA	ATG	1926/642	TGA	ATG	1659/553	TAA	ATG	1578/526	TGA	ATG	1044/348	TAG	ATG	1074/358	TGA	--	1035/345	TGA	ATG	690/230	TAA
DH13C120	ATG	3909/1303	TAA	ATG	2847/949	TAG	ATG	2694/898	TAA	ATG	1926/642	TGA	ATG	1659/553	TAA	ATG	1575/525	TGA	ATG	1044/348	TAG	ATG	1074/358	TGA	ATG	1035/345	TAA	ATG	699/233	TGA
YN15-283-01	ATG	3909/1303	TAA	ATG	2847/949	TAG	ATG	2694/898	TAA	ATG	1926/642	TGA	ATG	1659/553	TAA	ATG	1575/525	TGA	ATG	1044/348	TAG	ATG	1074/358	TGA	ATG	1038/346	TAA	ATG	699/233	TGA
V290/YNSZ	ATG	3909/1303	TAA	ATG	2763/921	TAA	ATG	2694/898	TAA	ATG	1926/642	TGA	ATG	1659/553	TAA	ATG	1578/526	TGA	ATG	1044/348	TAG	ATG	1074/358	TGA	ATG	1041/347	TAA	ATG	690/230	TAA
V298/YNJH	ATG	3909/1303	TAA	ATG	2760/920	TAG	ATG	2694/898	TAA	ATG	1926/642	TGA	ATG	1659/553	TAA	ATG	1578/526	TGA	ATG	1044/348	TAG	ATG	1074/358	TGA	ATG	1038/346	TAA	ATG	690/230	TAA
YNV/17-14	ATG	3909/1303	TAA	ATG	2826(942)	TAG	ATG	2694/898	TAA	ATG	1926/642	TGA	ATG	1659/553	TAA	ATG	1575/525	TGA	ATG	1044/348	TAG	ATG	1074/358	TGA	ATG	1038/346	TAA	ATG	690/230	TAA
YNV/KM-1	ATG	3909/1303	TAA	ATG	2763(921)	TAA	ATG	2694/898	TAA	ATG	1926/642	TGA	ATG	1659/553	TAA	ATG	1578/526	TGA	ATG	1044/348	TAG	ATG	1074/358	TGA	ATG	1041/347	TAA	ATG	690/230	TAA

1) IC: initiation codon
2) TC: termination codon

3) -: Missing initiation codon
